# Removing uncertainty from variants of unknown significance

**DOI:** 10.1172/JCI171497

**Published:** 2023-06-15

**Authors:** Elizabeth M. McNally

Interpretation of genetic testing results is complicated by the high degree of genetic variation present in every individual genome. Most inherited single-gene disorders are caused by rare gene variants, but it is often not possible to distinguish between rare pathogenic variants and rare benign variants using current classification systems. These classification systems rely heavily on comparing disease prevalence with the population frequency of genetic variants, both of which may be inadequately reported. This ambiguity can lead to genetic tests yielding results called variants of unknown significance (VUSs), and uncertain results can be frustrating for both health care participants and providers. Reliable functional assays of all variants in a gene and/or protein provide additional critical information to improve genetic interpretation.

Over 50% of patients with myopathy are found to have at least one VUS in a myopathy gene, complicating genetic diagnosis (1). A subtype of limb-girdle muscular dystrophy (LGMD) is caused by recessive mutations in the *SGCB* gene. *SGCB*, which encodes β-sarcoglycan, together with α-, γ-, and δ-sarcoglycan, forms a four-protein transmembrane complex (SGC) that stabilizes the muscle plasma membrane, and mutations in any of the sarcoglycan subunits can lead to LGMD (2, 3). Data that indicate native protein expression can aid in the genetic diagnosis for these muscle disorders, but gathering protein localization information requires an invasive muscle biopsy.

In this issue of the *JCI*, Li and colleagues assessed the impact of missense variants by performing deep mutational scanning of the *SGCB* gene and evaluating the cell-surface localization of the sarcoglycan complex for all 6,340 possible amino acid changes (4). The functional assay scored missense variants based on cell surface production of the sarcoglycan complex. For known pathological variants, the degree of cell surface production correlated with the clinical outcome of loss of ambulation. Variants with less severe functional scores often correlated with slower disease progression, demonstrating a link between variant function and disease severity. The authors used AlphaFold2 to identify domains in this single-pass type 2 transmembrane protein enriched for pathological variants. These findings can aid in the clinical interpretation of *SGCB* variants and enhance LGMD diagnosis, and, with clinical trials ongoing for β-sarcoglycan gene replacement therapy, accurate diagnosis is an essential step.

The predicted structural information in Li et al. not only identified regions in which pathogenic variants clustered, but described AlphaFold2 modeling that incorporated multiple sarcoglycan subunits alongside β-sarcoglycan that also helped identify interface residues and predicted pathogenicity of γ-sarcoglycan and δ-sarcoglycan residues (4). This work provides a clinically useful assay to interpret genetic variation in the sarcoglycan transmembrane complex, and this method can be extended to genes encoding other transmembrane proteins and cell-surface complexes (4).

Currently, clinical diagnosis of sarcoglycan-deficient LGMD is challenging owing to the overlap in phenotype between different sarcoglycanopathies and the phenotypic heterogeneity of other genetically defined LGMDs. Obtaining a genetic diagnosis can help determine genotype-phenotype correlations and provide useful interpretation for patients and families.

## Version 1 06/15/2023

Electronic publication
